# Carboplatin-induced hematotoxicity among patients with non-small cell lung cancer: Analysis on clinical adverse events and drug-gene interactions

**DOI:** 10.18632/oncotarget.12951

**Published:** 2016-10-27

**Authors:** Yi-ju Cheng, Ran Wu, Ming-liang Cheng, Juan Du, Xi-wei Hu, Lei Yu, Xue-ke Zhao, Yu-mei Yao, Qi-zhong Long, Li-li Zhu, Juan-juan Zhu, Ni-wen Huang, Hua-juan Liu, Ya-xin Hu, Fang Wan

**Affiliations:** ^1^ Department of Respiratory Medicine, The Affiliated Hospital of Guizhou Medical University, Guiyang, Guizhou, China; ^2^ Department of Respiratory Medicine, The Affiliated Baiyun Hospital of Guizhou Medical University, Guiyang, Guizhou, China; ^3^ Department of Dermatology, The First Affiliated Hospital of Guiyang college of Traditional Chinese Medicine, Baoshanbei Guiyang, Guizhou, China; ^4^ Department of Infectious Diseases, The Affiliated Hospital of Guizhou Medical University, Guiyang, Guizhou, China; ^5^ Prenatal Diagnostic Center, The Affiliated Hospital of Guizhou Medical University, Guiyang, Guizhou, China

**Keywords:** carboplatin, anemia, neutropenia, thrombocytopenia, FDA adverse event reporting system

## Abstract

In order to clarify the risk of hematotoxicity of carboplatin, we inspected 19901 case reports of non-small cell lung cancer patients that were submitted to the FDA Adverse Event Reporting System (FAERS) between January 2004 and December 2015. These comprised 3907 cases which were treated with carboplatin and 15994 cases which were treated with other therapies in the absence of carboplatin. By comparison, carboplatin cases were significantly more likely to report anemia (OR = 2.27, 95% CI 1.85-2.78, *P* = 5.04×10^−15^), neutropenia (OR = 2.27, 95% CI 1.76-2.92, *P* = 2.39×10^−10^), and thrombocytopenia (OR = 2.38, 95% CI 1.84-3.08, *P* = 5.60×10^−11^). We further explored published evidences and found 205 human genes interacting with carboplatin. Functional analysis corroborated that these genes were significantly enriched in the biochemical pathway of hematopoietic cell lineage (adjusted *P* = 6.02×10^−11^). This indicated that carboplatin could profoundly affect the development of blood cells. Given the early awareness of the hematologic risks, great caution should be exercised in prescribing carboplatin to non-small cell lung cancer patients. And functional enrichment analysis on carboplatin-related genes warranted subsequent research with regard to the underlying toxicological mechanisms.

## INTRODUCTION

As a platinum coordination compound, carboplatin is broadly used as a chemotherapeutic agent to treat various types of cancer, such as non-small cell lung cancer [1]. Carboplatin interferes with DNA repair, so as to suppress and eventually kill cancer cells [2]. However, the cell growth of normal tissues may also be affected by carboplatin [3]. As a result, serious adverse effects occur in the course of carboplatin-based chemotherapy. For example, the FDA-approved drug label [4] and a series of clinical studies [5, 6] have warned of the association between carboplatin and hematotoxicity, including anemia, neutropenia and thrombocytopenia (the deficiency of erythrocytes, neutrophils and platelets, respectively).

However, it should be noticed that hematotoxicity is not exclusive to carboplatin. As common hematologic complications of chemotherapy, anemia, neutropenia and thrombocytopenia have been widely observed in cancer patients treated with various drugs [7–9]. Although non-comparative studies have reported on the safety risks of carboplatin, there is still an urgent need for comparative case-control study to directly compare carboplatin with other anticancer therapies. If the incidence of hematologic disorders is relatively lower among carboplatin cases, there will be good reasons to reassess the safety profile of carboplatin. Otherwise, if carboplatin is significantly more likely to induce hematotoxicity, this drug must be used even more carefully. Such information will provide important references for clinicians to improve drug selection and drug dosing for the treatment of non-small cell lung cancer.

Such comparative study requires adverse event data of large samples, which is one of the major advantages of FDA post-market safety monitoring [10]. FDA Adverse Event Reporting System (FAERS) is a post-market surveillance system built up by US FDA to monitor the safety risks of approved drug products. Drug adverse events are spontaneously reported by healthcare professionals and patients to FAERS. Every report is described with a series of indexes of clinical information, including the disease(s) the patient had, the drug(s) used by the patient and the adverse reaction(s) that were observed. According to these indexes, a set of reports with certain characteristics can be extracted for analysis. Apart from drug safety surveillance of FDA, FAERS also supported a great deal of academic research on drug safety and development in recent years [11–13]. Given the increasingly extensive application of FAERS data, FDA launched the openFDA initiative (https://open.fda.gov/) in June 2014, which enabled researchers to efficiently download and preprocess computer-readable information [14]. In that way, the reporting rate of a certain adverse effect can be compared between different drugs.

The present study aimed to determine whether there were differences in hematologic risks between the cases treated with carboplatin and those not exposed to carboplatin. We inspected adverse event reports of non-small cell lung cancer patients that were submitted to the FAERS between January 2004 and December 2015. The results showed that hematologic adverse events were significantly more reported among carboplatin cases. In addition, we explored published evidences and found a set of human genes interacting with carboplatin. Functional enrichment analysis on these genes explained the potential toxicological mechanisms of carboplatin-induced hematotoxicity.

## RESULTS

### Pooled Analysis of hematotoxicity adverse events

The current meta-analysis on hematotoxicity involved a total of 19901 adverse events of non-small cell lung cancer patients, which were comprised of 3907 cases exposed to carboplatin and 5994 controls treated with other therapies in the absence of carboplatin. For each year between 2004 and 2015, a reporting odds ratio (ROR) [15] regarding hematologic adverse events was calculated by comparing the carboplatin cases with the controls (see Materials and Methods). And the meta-analysis pooling individual RORs together led to an overall estimate.

**Figure 1:**
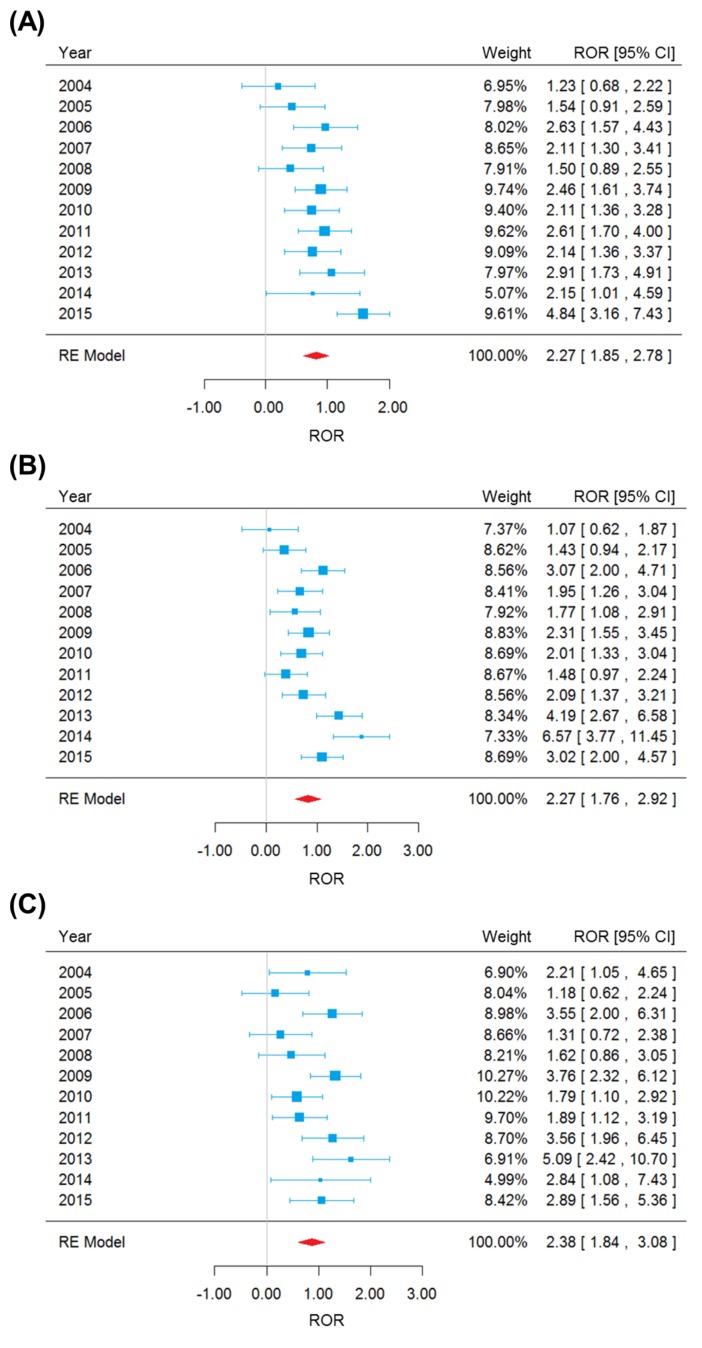
Forest plot of random-effects (RE) meta-analysis on anemia **A**., neutropenia **B**. and thrombocytopenia **C**. adverse events.

**Figure 2:**
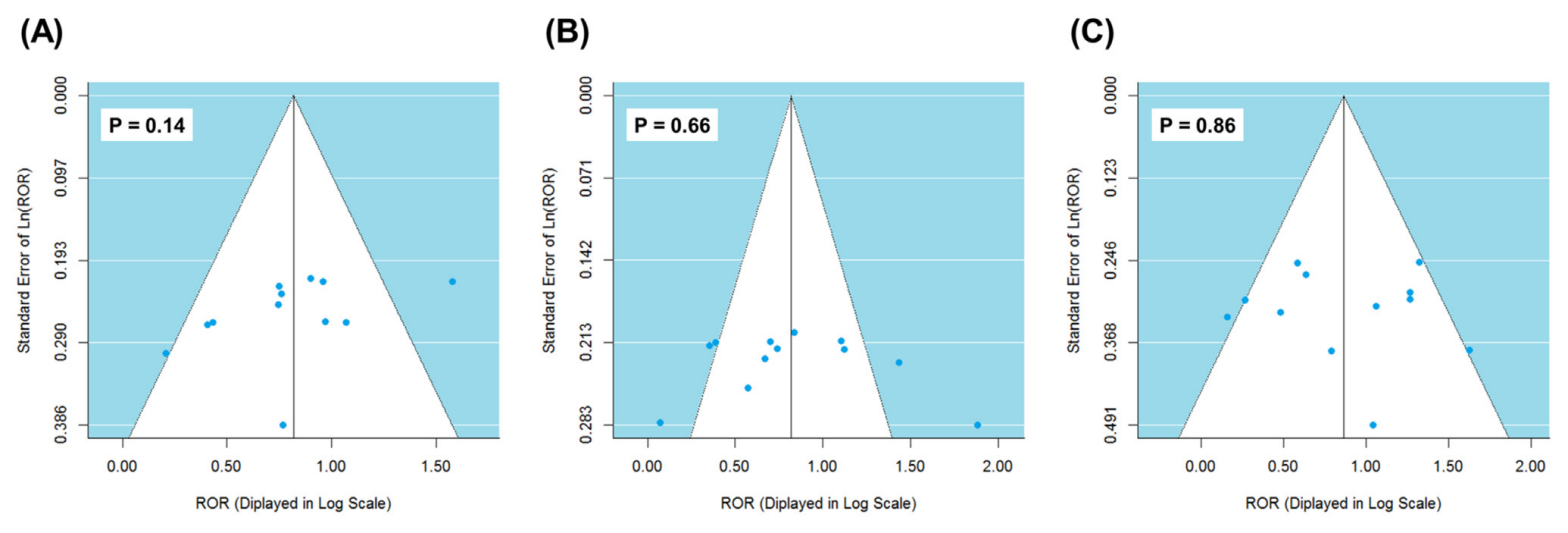
Funnel plot for detecting reporting bias of anemia **A**., neutropenia **B**. and thrombocytopenia **C**. adverse events.

We performed χ^2^-based Q test and calculated I^2^ statistics to examine the heterogeneity underlying the FAERS reports of different years [16]. There was significant heterogeneity for anemia, neutropenia and thrombocytopenia (Table 1), which justified the random-effects model for meta-analysis (see Materials and Methods). Overall, significantly higher risks of hematotoxicity were observed in carboplatin cases (Figure 1). Carboplatin was more often reported for anemia (pooled ROR = 2.27, 95% CI 1.85-2.78, *P* = 5.04×10^−15^), neutropenia (pooled ROR = 2.27, 95% CI 1.76-2.92, *P* = 2.39×10^−10^), and thrombocytopenia (pooled ROR = 2.38, 95% CI 1.84-3.08, *P* = 5.60×10^−11^). Funnel plot was generated and Egger's linear regression test [17] was performed to evaluate the reporting bias of adverse events (see Materials and Methods). The shape of funnel plot did not reveal any evidence of detectable asymmetry (Figure 2) and suggested the absence of reporting bias. Leave-one-out sensitivity analyses (i.e., a single year in the meta-analysis was deleted each time to observe the influence on the value of pooled ROR) showed that the lower bounds of 95% CI of ROR were constantly higher than 1.00 (data not shown). These tests corroborated that the positive results were reliable and robust.

**Table 1:** Result of Q test for heterogeneity between different years

Adverse Effect	*P*-value of Q test	I^2^ statistics (%)	Method of Meta-analysis
Anemia	0.02	51.32	Random-effects Model
Neutropenia	1.26×10^−5^	75.20	Random-effects Model
Thrombocytopenia	0.015	53.15	Random-effects Model

### Analysis on genes interacting with carboplatin

**Figure 3:**
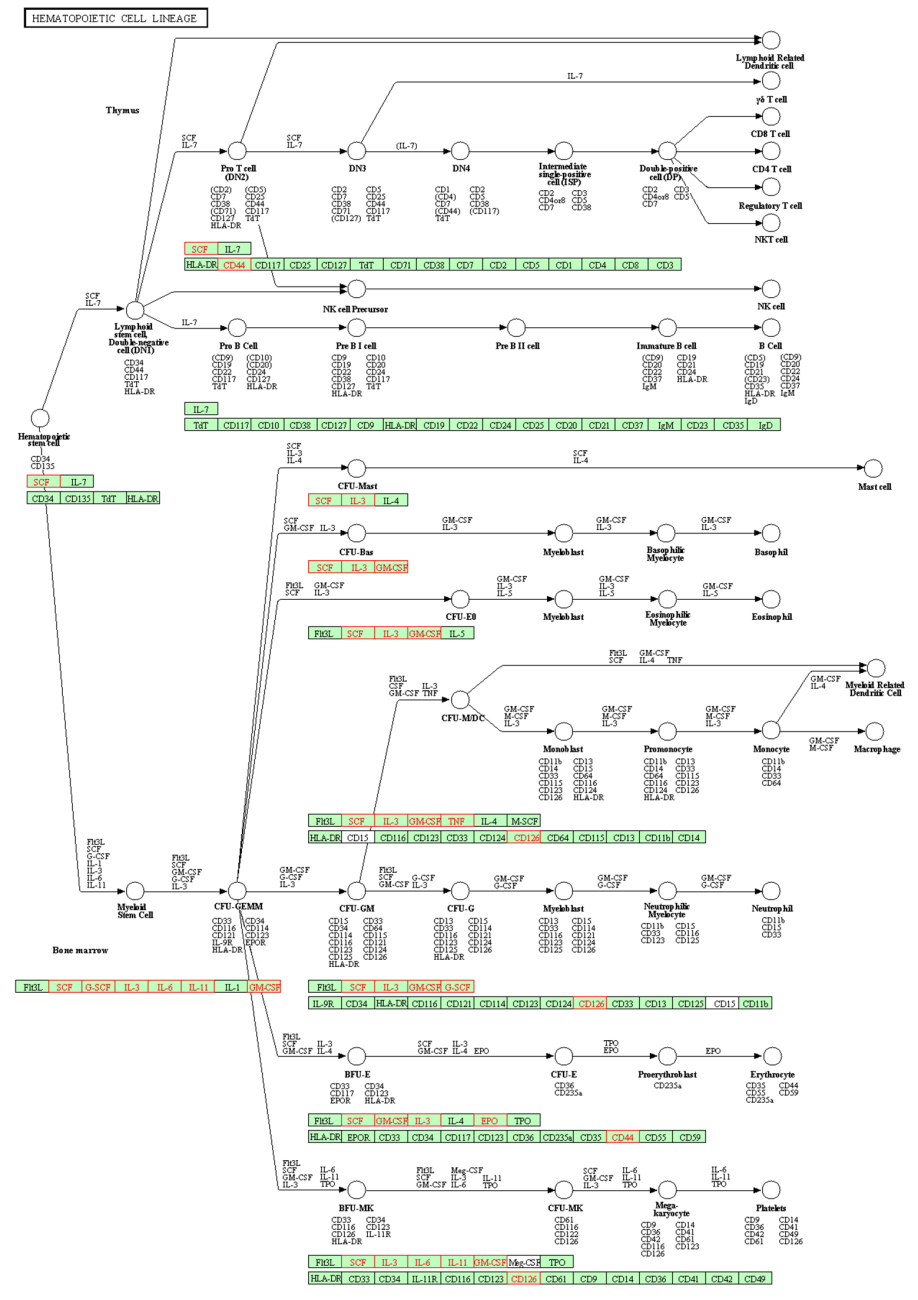
Visualization of carboplatin-related genes (highlighted in red color) enriched in the hematopoietic cell lineage KEGG pathway

It is a well-known fact that drug effects on human body work through the interaction between the drug molecule and various genes/proteins [18]. Therefore, adequate exploration of drug-gene relation can greatly facilitate understanding the toxicological mechanisms of carboplatin-induced hematotoxicity. To address this need, we searched the Drug2Gene knowledge base [19] and efficiently identified a set of 205 human genes interacting with carboplatin (Supplementary Data S1). The relation between each of these genes and carboplatin was supported by published and/or experimentally tested evidences derived from 23 source databases (see Materials and Methods). These genes served as starting points to explain the therapeutic and adverse effects of carboplatin. To interpret the biological functions of this gene set, we further queried the WebGestalt (WEB-based GEne SeT AnaLysis Toolkit) online server [20] and performed enrichment analysis for the KEGG pathways [21] (see Materials and Methods; Table 2 and Supplementary Data S2). First, compared with the background distribution of human genome, the carboplatin-related genes were unsurprisingly enriched in a series of pathways involved in non-small cell lung cancer and other types of cancer, suggesting that the drug-gene relation data could effectively characterize the mechanism of action of carboplatin. Besides, we considered non-cancer pathways and found strong perturbations in hematopoietic cell lineage, i.e., the development progresses of various blood cells (Supplementary Data S3). Carboplatin was found to interact with a number of genes encoding cytokine (CSF2, CSF3, EPO, IL11, IL3, IL6 and TNF), cytokine receptor (IL6R), ligand of tyrosine-kinase receptor (KITLG) and cell-surface glycoprotein (CD44), which played fundamental roles in the formation of erythrocytes, neutrophils and platelets (Figure 3). While only 88 genes in human genome belonged to the pathway of hematopoietic cell lineage, 10 out of the 205 carboplatin-related genes fell in the category. Such a significant enrichment (enrichment ratio = 24.02, adjusted *P* = 6.02×10^−11^) showed that carboplatin profoundly affect the development of blood cells by interrupting certain key genes.

**Table 2:** A portion of KEGG pathways showing significant enrichment of carboplatin-related benes

KEGG Pathway	Number of Human Genes in the Category	Number of Carboplatin-related Genes in the Category	Enrichment Ratio	Adjusted *P*-value
Pathways in cancer	326	43	27.88	4.48×10^−47^
Colorectal cancer	62	17	57.97	3.03×10^−24^
Small cell lung cancer	85	16	39.79	4.45×10^−20^
Prostate cancer	89	16	38.01	8.42×10^−20^
Chronic myeloid leukemia	73	14	40.54	6.77×10^−18^
Pancreatic cancer	70	13	39.26	1.52×10^−16^
Bladder cancer	42	9	45.3	2.41×10^−12^
Non-small cell lung cancer	54	9	35.23	1.98×10^−11^
Hematopoietic cell lineage	88	10	24.02	6.02×10^−11^
Glioma	65	9	29.27	1.00×10^−10^
Melanoma	71	9	26.8	2.09×10^−10^
Renal cell carcinoma	70	8	24.16	4.87×10^−09^
Acute myeloid leukemia	57	7	25.96	2.81×10^−08^
Thyroid cancer	29	3	21.87	0.0005

## DISCUSSION

Anticancer chemotherapies frequently induce bone marrow suppression and consequent deficiency of blood cells in cancer patients [22]. In some cases, the deficiency of blood cells may lead to life-threatening outcomes, such as severe infections [23] and bleeding disorders [24]. Furthermore, cancer patients affected by chemotherapy-induced hematotoxicity are usually subject to a substantial economic burden in terms of medical costs [25]. To address these problems, there is an urgent need to identify risk factors, such as the drugs more liable to hematotoxicity and the genes involved in the toxicological mechanisms. The main objective of the present study was to elucidate the comparative hematologic risks of carboplatin and to find clues to the toxicology of carboplatin.

As a derivative of anticancer drug cisplatin, carboplatin gained increasing acceptance in clinical treatment for reduced non-hematologic toxicity [1]. On the other hand, carboplatin was observed with greater risk of bone marrow suppression than cisplatin [26]. However, there has been a long lack of large-scale case-control studies that directly compare the hematologic risks between carboplatin treatment and non-carboplatin anticancer therapies. Here we retrieved large samples of non-small cell lung cancer patients from FAERS, which enabled more efficient detection of safety signals. The result demonstrated that carboplatin cases were significantly more likely to report anemia, neutropenia and thrombocytopenia than non-carboplatin controls. Early awareness of the hematologic risks of carboplatin will facilitate a rational clinical management of cancer patients [27]. As in many studies on post-market safety data, some caveats still need to be considered. For example, dose reduction is a common practice in chemotherapy for cancer treatment to control the risk of toxicity [28, 29]. However, due to the spontaneous nature of FAERS reports, detailed information about drug dosage could not be guaranteed in many records. We therefore expect subsequent research to investigate how the dosage factor [30] may influence carboplatin-induced hematotoxicity. In addition, carboplatin is used to treat not only non-small cell lung cancer but also many other types of cancer [31, 32]. Given the adverse events observed in non-small cell lung cancer patients, it should be reasonable to further investigate carboplatin-induced hematotoxicity in other pathological conditions. Another concern is the varying quality of spontaneous reports in FAERS and the confounding effects in data analysis (e.g., age, comorbidities, and prior treatment history) [33], which requires further research based on different clinical samples.

Chemotherapy-induced hematotoxicity is mainly relieved with either transfusion of blood cells or administration of hematopoietic growth factors [34]. However, without fully understanding the underlying toxicological mechanisms, that can only fix the symptom rather than the root cause. Therefore, understanding the toxicology of carboplatin as the basis of prevention is an important research priority. Based on the drug-gene relation information from public databases, we investigated a set of 205 human genes interacting with carboplatin, some of which belonged to the KEGG pathway of hematopoietic cell lineage. For example, proteins encoded by CSF2 and CSF3 have been used experimentally and clinically to treat chemotherapy-induced myelosuppression [35, 36]. And interleukins have been proved to be associated with hematologic complications [37, 38]. These evidences supported the notion of explaining drug action by the biological functions of drug-related genes [39]. Therefore, other carboplatin-related genes involved in hematopoietic cell lineage may provide new research routes with regard to the underlying toxicological mechanisms of carboplatin, such as finding polymorphisms associated with hematotoxicity [40] and differential gene expression involved in hematopoietic lineage [41]. Experimental validation of these genes will be required to further elucidate the roles they play in the hematologic context.

In summary, hematological adverse events were significantly more reported by carboplatin cases than controls receiving other anticancer therapies, suggesting that great caution should be exercised in prescribing carboplatin to non-small cell lung cancer patients. This result had implications for clinical management of cancer patients and prophylaxis of hematotoxicity. And the genes interacting with carboplatin were found to be significantly enriched in the biochemical pathway of hematopoietic cell lineage, which warranted subsequent toxicological research.

## MATERIALS AND METHODS

### Extraction of raw adverse events

The original adverse events were queried according to the official tutorial of openFDA platform (https://open.fda.gov/api/reference/). Two investigators independently queried the data and the results were reviewed by a third investigator. Inconsistency was solved by discussion with the whole research team. 19901 adverse events of non-small cell lung cancer patients submitted between 2004 and 2015 were extracted by searching in the drug indication index for “NON-SMALL CELL LUNG CANCER”. Among these adverse events, those related to carboplatin were specified with the drug generic name “CARBOPLATIN”. All reported adverse events in FAERS were coded using Medical Dictionary for Regulatory Activities (MedDRA) terminology (http://www.meddra.org/). So anemia, neutropenia and thrombocytopenia events were specified by searching in the adverse effect index for “ANAEMIA”, “NEUTROPENIA” and “THROMBOCYTOPENIA”, respectively.

### Statistical Analysis

We examined the association between the carboplatin and hematotoxicity. For each year between 2004 and 2015, a two-by-two contingency table was constructed, in which subjects were classified by carboplatin exposure (exposed or not exposed) and hematologic adverse effect (reported or not reported). Reporting odds ratio (ROR) and its 95% confidence interval (95% CI) were calculated to assess the strength of the association. An ROR significantly greater than 1.00 indicated a higher risk of hematotoxicity for carboplatin.

Then, the data of different years were pooled together to estimate the overall ROR. Such a meta-analysis was performed with the ‘metafor’ package (https://cran.r-project.org/web/packages/metafor/) of R software. The heterogeneity between different years was assessed with the χ^2^-based Q test, and a *P*-value < 0.05 indicated significant heterogeneity. Meanwhile, I^2^ = 100% × (Q - df)/Q) was calculated as another statistics to measure the proportion of total heterogeneity contributed by between-year variation. When significant heterogeneity was observed, the pooled ROR was calculated by a random-effects model (the DerSimonian and Laird method). Otherwise, a fixed-effects model (the Mantel-Haenszel method) was selected.

Additionally, in case of bias that may be introduced in the process of reporting adverse events, we used the funnel plot to assess the validity of meta-analysis. The asymmetry of funnel plot was assessed with Egger's linear regression test. A statistically significant asymmetry (*P*-value < 0.05) was considered as reporting bias.

### Drug-gene interactions

The information of relations between carboplatin and genes/proteins was retrieved from Drug2Gene (http://www.drug2gene.com), a freely accessible knowledge base that combined data from 23 public databases to provide a ‘one-stop shop’ for finding all genes related to a certain drug. By searching in the compound name index for ‘carboplatin’ and in the organism name index for ‘Homo sapiens’, a set of 205 human genes were found to interact with carboplatin’. Then, the gene set was input into the WEB-based GEne SeT AnaLysis Toolkit (WebGestalt, http://bioinfo.vanderbilt.edu/webgestalt/) to perform hypergeometric test and evaluate the enrichment for the KEGG pathways. As multiple pathways were tested at the same time, the p-values of enrichment were adjusted using the Benjamini-Hochberg procedure. Pathways with adjusted *P*-value < 0.01 were selected due to statistically significant enrichment.

## SUPPLEMENTARY MATERIALS TABLES






